# Supplementary material for: MEMS enabled miniaturized light-sheet microscopy with all optical control
Source: Sci Rep. 2021 Jul 8;11:14100. doi: 10.1038/s41598-021-93454-8 (PMC8266809; doi:10.1038/s41598-021-93454-8)
Supplement: Supplementary file 1 — Supplementary Informations. [file 41598_2021_93454_MOESM1_ESM.docx]

**MEMS enabled miniaturized light-sheet microscopy with all optical control – supplementary material**

**Spyridon Bakas, Deepak Uttamchandani, Hiroshi Toshiyoshi, and Ralf Bauer**

The supplementary material document covers three parts in addition to the main manuscript:

Supplementary Fig. 1: A comparison of the image quality and aberration reduction introduced by the use of the 3D-printed prism, comparing sub-resolution bead images of a system with and without the prism.

Supplementary Fig. 2: A comparison of maximum intensity projections of a cell slide sample using the microscope coordinate system xyz and the sample coordinate system x’y’z’.

Supplementary Table 1: A list of all components used for the microscope with current price estimates.

**Supplementary Fig. 1, Astigmatism reduction through 3D-printed prism**


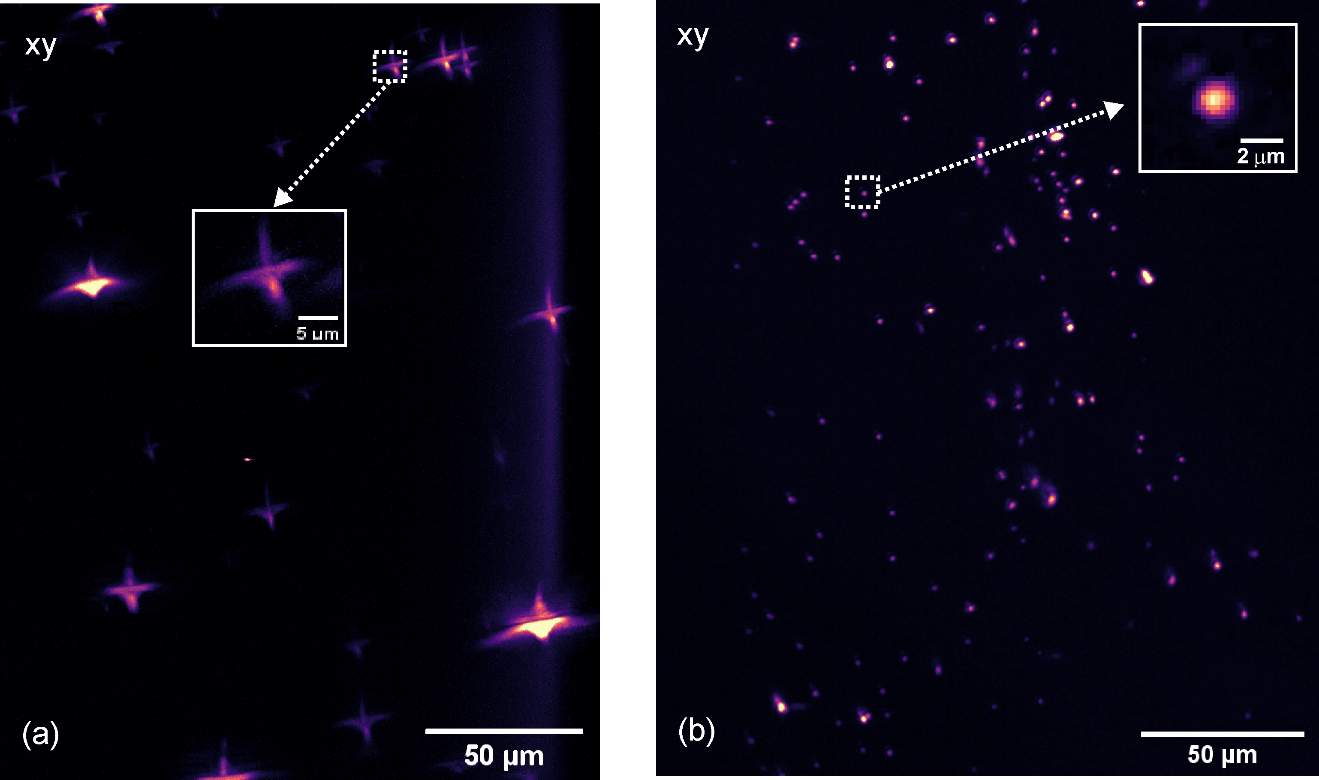


Comparison of nano-bead images with and without the inclusion of the 3D-printed prism. (a) xy maximum intensity projection of the system without the prism showing astigmatism originating from the 30° angled imaging of the cover-slip mounted sample. (b) xy maximum intensity projection of the system as presented in the manuscript, including the 3D-printed prism.

**Supplementary Fig. 2, Cell slide images in microscope coordinate frame and sample coordinate frame**


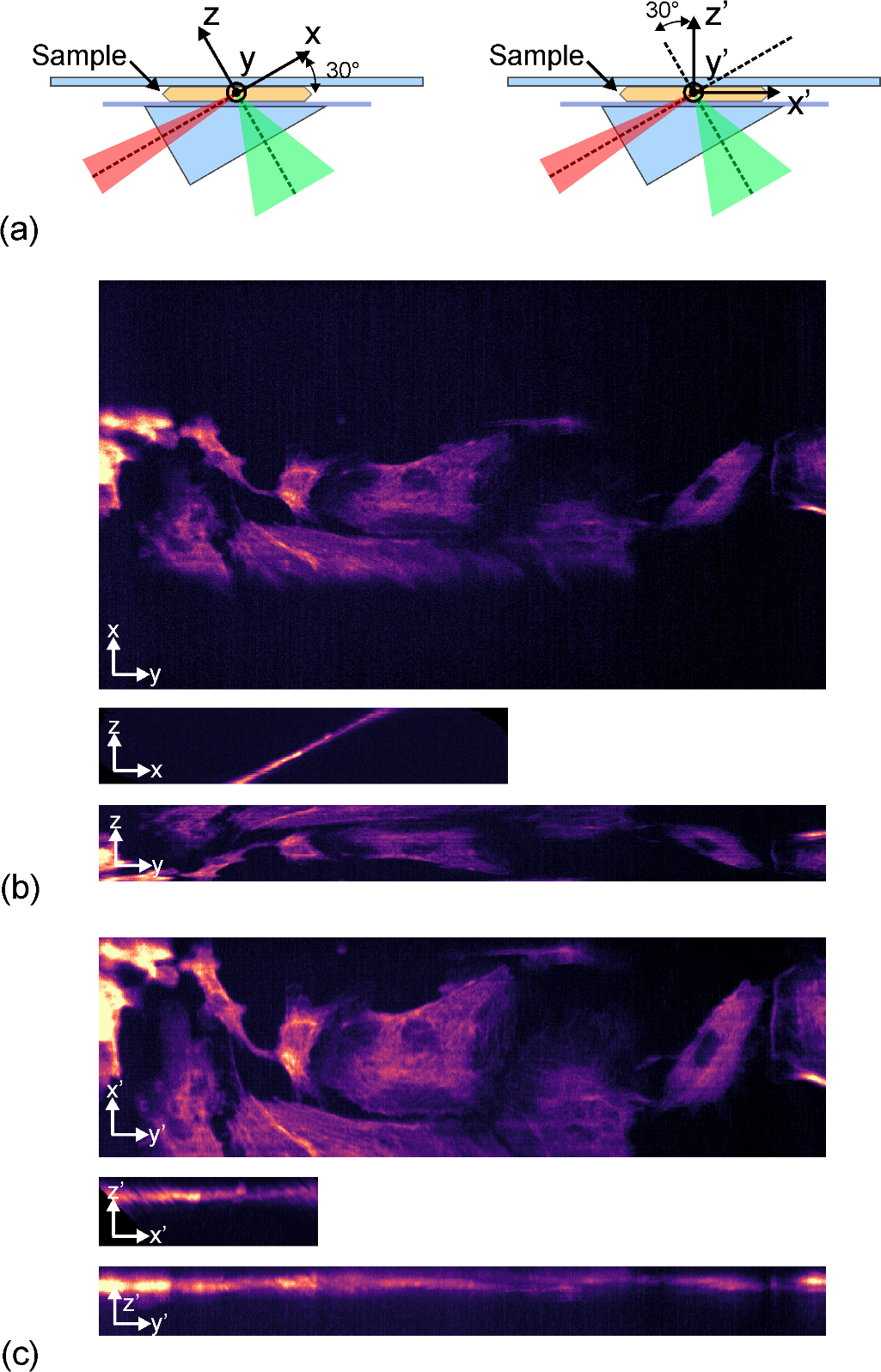


Comparison of maximum intensity projections of a BPAE cell slide shown in the microscope coordinate system and sample coordinate system. (a) Coordinate system definition. (b) Maximum intensity projection in xy, xz and yz in the microscope coordinate system. (c) Maximum intensity projection in x’y’, x’z’ and y’z’ in the sample coordinate system.

**Supplementary Table 1, List of materials for Miniaturised LSM system**

| **Item** | **Manufacturer** | **Manufacturer number** | **Cost (£)** |  |
| --- | --- | --- | --- | --- |
| Breadboard | Thorlabs | MB2530/M | 107 |  |
| Single mode fibre | Thorlabs | S405-XP-Custom | 125 |  |
| Fibre collimator | Thorlabs | CFC-2A | 216 |  |
| Adapter for collimator | Thorlabs | AD15F2 | 23 |  |
| Lens holder | Thorlabs | LMR1/M | 12 |  |
| 1" post, 3.5" height | Thorlabs | RS3.5P/M | 26 |  |
| 1" post, 1.5" height | Thorlabs | RS1.5P/M | 20 |  |
| 1" post, 2" height | Thorlabs | RS2P/M | 22 |  |
| 1" post clamps x3 | Thorlabs | CF125 | 21 |  |
| 50mm post x2 | Thorlabs | TR50/M | 8 |  |
| 100mm post | Thorlabs | TR100/M | 5 |  |
| post holder x2 | Thorlabs | UPH40/M | 49 |  |
| 1/2" mirror | Thorlabs | ME05/P01 | 13 |  |
| 1/2" mirror holder | Thorlabs | KM05/M | 30 |  |
| 90deg post braket | Thorlabs | RA90/M | 8 |  |
| 3-axis stage | Newport | M-DS25-XYZ | 258 |  |
| Adapter for 3-axis stage | Newport | M-B-2C | 35 |  |
| 3-axis stage x2 | Thorlabs | DT12XYZ | 492 |  |
| 1/2" mirror holder | Thorlabs | KM05/M | 30 |  |
| 1/2" lens tube | Thorlabs | SM05L20C | 43 |  |
| 1/2" Lens tube holder | Thorlabs | SM05TC | 32 |  |
| Adapter plates x2 | Thorlabs | RS5M | 12 |  |
| 1" lens tube | Thorlabs | SM1L40 | 35 |  |
| 1" lens tube holder | Thorlabs | SM1TC | 34 |  |
| 7.5mm lens x2 | Thorlabs | AC050-008-A | 73 |  |
| 30mm lens | Thorlabs | AC127-030-A | 41 |  |
| x20 objective | Newport | MVC-20X | 98 |  |
| RMS to 1" adapter | Thorlabs | SM1A3 | 14 |  |
| tunable lens | Edmund Optics | 37-344 | 106 |  |
| Emission Filter | Thorlabs | FEL0500 | 60 |  |
| Camera | Thorlabs | CS2100M-USB | 2379 |  |
| 3D-printed prism | Formlabs | custom | 5 |  |
| 3D-printed adapter | Formlabs | custom | 25 |  |
| MEMS | Stanley Inc | custom | 85 |  |
| Arduino Due | Arduino | Due | 31 |  |
| Drive electronics | custom | custom | 100 |  |
|  |  | overall | 4673 | (ex VAT) |
